# Pose-Perceptive Convolution: Learning Geometry-Adaptive Receptive Fields for Robust 6D Pose Estimation

**DOI:** 10.3390/s26020453

**Published:** 2026-01-09

**Authors:** Yi Lai, Yaqing Song, Qixian Zhang, Yue Wang, Kang An, Hui Zhang

**Affiliations:** 1 College of Information, Mechanical and Electrical Engineering, Shanghai Normal University, Shanghai 201418, China; willow984@outlook.com (Y.L.); yqsong@shnu.edu.cn (Y.S.); 1000549399@smail.shnu.edu.cn (Y.W.); 2 School of Computer Science and Technology, Tongji University, Shanghai 201804, China; zhangqx@tongji.edu.cn

**Keywords:** 6D pose estimation, RGB-depth fusion, geometric mismatch, receptive-field adaptation

## Abstract

6D object pose estimation is crucial for applications such as robotic manipulation and augmented reality, yet it remains highly challenging when dealing with objects of significantly different aspect ratios or the drastic appearance variations of a single object caused by pose changes. Most existing methods focus on designing more complex backend fusion modules, while largely overlooking a fundamental problem at the feature extraction frontend: the geometric mismatch between the fixed, square receptive fields of standard convolutions and the varied projected morphologies of objects. This mismatch, along with noise in fused features and ambiguity in regression, limits the performance ceiling of current methods. To this end, this paper proposes a novel Pose-Perceptive Convolution (PPC) and constructs a new Pose-Perceptive Fusion Network (PPF-Net). Its core component, the Pose-Perceptive Convolution, fundamentally resolves the aforementioned geometric mismatch by dynamically adapting the shape and sampling density of its receptive field. Experiments on four benchmarks show that PPF-Net improves the VSD score by 19.4% over FFB6D on MP6D, and achieves 96.7% ADD-S on YCB-Video, approaching state-of-the-art accuracy. Crucially, these gains are realized with minimal computational overhead, avoiding the heavy latency of backend-intensive approaches. This validates that frontend feature extraction is an efficient strategy for robust 6D pose estimation.

## 1. Introduction

6D object pose estimation is a fundamental task that recovers the 3D rotation and translation of known objects relative to a sensor. This technology is a critical component for applications such as robotic grasping, automated assembly, and augmented reality [1]. Among the available methods, the single-frame, single-view RGB-D method is a mainstream engineering solution. This approach is favored for its operational simplicity, as it avoids the complexities of multi-view systems or temporal information processing.

Early 6D object pose estimation methods primarily relied on geometric or template-matching techniques. Whether based on template matching [2] or voting schemes using local geometric descriptors [3,4], these approaches are highly dependent on handcrafted features, which makes them fragile when facing real-world complexities such as varying illumination, heavy occlusion, and symmetric or textureless surfaces [5]. This spurred the field’s transition towards deep learning methods capable of learning data-driven feature representations.

On one hand, RGB-based deep learning methods [6,7] excel in texture-rich scenarios by learning dense 2D-to-3D correspondences, but their stability is limited when handling occlusion and textureless surfaces due to the lack of explicit depth constraints. On the other hand, deep learning methods that leverage depth information [8] operate directly in 3D space, exhibiting stronger robustness to occlusion, yet they cannot utilize texture to distinguish between geometrically similar objects. To combine the advantages of both single modalities, RGB-D fusion methods, from DenseFusion [9] to FFB6D [10] and RCVPose [11], emerged and became a mainstream solution. However, their RGB branches still rely on standard convolutions with fixed, square receptive fields. Given that a 3D pose change induces large variations in the shape and scale of the 2D projection, this rigid sampling grid creates a geometric mismatch with the object’s appearance. This mismatch introduces a foundational bias at the earliest stage of feature extraction, meaning that even the most powerful subsequent fusion mechanisms are fundamentally constrained to mending a distorted representation.

To further push the performance boundaries, two main technical trends centered on enhancing backend processing capabilities have emerged. The first is the “render-and-compare” trajectory [12,13], which improves accuracy by explicitly optimizing for visible surface consistency. However, such methods often entail high computational costs and engineering complexity, and are heavily dependent on high-fidelity rendering pipelines, posing challenges for practical deployment. The second trajectory involves the introduction of cross-modal Transformers or heavy attention mechanisms [14]. Although these powerful backend modules can improve performance, they significantly increase the model’s computational and memory requirements and cannot compensate for the geometric mismatch introduced by the initial standard convolutions. Furthermore, the underutilization of predicted uncertainty is another common limitation across various learning-based methods [15]. Although some works have used uncertainty to guide loss weighting during training, most approaches still output a single, deterministic pose estimate, making it difficult to effectively handle the inherent pose ambiguities caused by symmetries or heavy occlusion.

In summary, while existing technical branches have all made significant contributions, they share several key unaddressed gaps: first, the mismatch between frontend feature sampling and the object’s projected morphology remains unresolved; second, a lightweight mechanism to suppress noise in fused features is lacking; and finally, most approaches still output a single, deterministic pose estimate, making it difficult to effectively handle the inherent pose ambiguities caused by symmetries or heavy occlusion.

**Figure 1:**
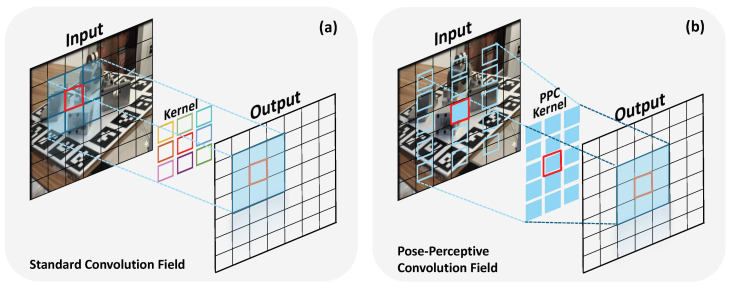
Comparison between the receptive fields of (**a**) standard convolution and (**b**) the proposed Pose-Perceptive Convolution (PPC). Unlike the fixed, square receptive field of a standard convolution, the PPC dynamically adapts both its shape and sampling density to align with the geometric morphology of the object’s projection on the image plane.

To address the aforementioned challenges, this paper presents a different approach. Instead of continually increasing model complexity at the fusion backend, this work returns to the frontend of feature extraction, focusing on fundamentally resolving the geometric mismatch caused by object pose variations. In addition, the method refines fused features through a lightweight attention mechanism and effectively handles pose ambiguities via probabilistic regression. To clearly validate these methods, FFB6D was selected as the baseline, in contrast to more complex methods that depend on heavy backend such as Transformers or render-and-compare pipelines. The main contributions of this paper are summarized as follows:

- As shown in Figure 1, a novel Pose-Perceptive Convolution is proposed to resolve the geometric mismatch at the feature extraction frontend. Unlike standard convolutions or existing deformable convolutions that lack geometric priors, PPC simultaneously adapts the receptive field’s aspect ratio and sampling density to align with the object’s projected morphology.
- A lightweight post-fusion attention mechanism is introduced by adapting the parameter-free SimAM module. This integration is specifically designed to denoise fused features in occluded and reflective scenes, enhancing robustness with negligible computational overhead.
- A probabilistic regression strategy based on the Gaussian negative log-likelihood loss is introduced to model predictive uncertainty. By modeling predictive uncertainty, this mechanism enables the network to produce reliable pose estimates in ambiguous scenarios involving symmetries and occlusions, where deterministic regression often fails.
- Comprehensive experiments are conducted on multiple challenging benchmarks, showing that the proposed method achieves accuracy approaching current state-of-the-art methods while maintaining superior computational efficiency, validating the effectiveness of our frontend-focused design.

The remainder of this paper is organized as follows: Section 2 reviews related work. Section 3 details the proposed method. Section 4 presents the experimental results, and Section 5 concludes the paper.

## 2. Related Work

This section reviews the progression of 6D object pose estimation from early geometric methods to current deep learning paradigms, including RGB-D fusion, advanced backend strategies, and uncertainty modeling techniques.

### 2.1. From Geometry and Templates to RGB-D Fusion

Early 6D pose estimation relied on handcrafted features and geometric matching. Template matching typified by LINEMOD gave a practical solution for real-time estimation of textureless objects through gradient and surface normal cues [2]. Point Pair Features recovered poses from local geometric relations in point clouds and established a voting-based method family [3,4]. Early learning methods, for instance, by predicting object coordinates per pixel [16] or the 2D projections of bounding box corners [17], solved the pose with RANSAC and PnP, which reduced dependence on templates [18]. These methods are interpretable and easy to deploy with stable results under mild occlusion and distinct features. However, they remain sensitive to illumination, texture and heavy occlusion, and they often fail for symmetric objects, which motivated deep learning methods.

Deep learning first advanced along two single-modality lines. RGB-only methods recovered pose from images by direct regression and then by correspondences. PoseCNN pioneered end-to-end regression, PVNet introduced pixel-wise voting to object keypoints, CDPN predicted dense object coordinate maps for robust PnP [6,7,19,20,21]. These methods work well with rich texture but lack explicit depth constraints, which limits stability under scale ambiguity, specular reflection and heavy occlusion [22,23]. Point-cloud-dominant methods operated in 3D. PointFusion combined geometric features with image features. PVN3D performed keypoint voting directly on point clouds, inspired by 3D detection methods like VoteNet [24], and improved robustness to occlusion [8,25]. However, the absence of texture cues hampers discrimination between geometrically similar objects, and the quality of the point cloud constrains performance [26,27]. These limitations of single-modality methods indicate the need for RGB-D fusion.

RGB-D fusion combines the strengths of single-modality pipelines and has become mainstream. DenseFusion introduced dense fusion of image features and geometric features at the point level and established a strong baseline for later research [9]. FFB6D advanced this idea with bidirectional information exchange at each stage of the encoder–decoder, which strengthened interaction between the image branch and the depth branch [10]. RCVPose further improved the voting process with a rotational consistency constraint and reported state-of-the-art results on multiple benchmarks [11,28]. While such direct-prediction frameworks became dominant, other methods like particle filtering were also explored for robust fusion [29]. Recent approaches such as RDPN6D [30] and CMAGCA [31] continue to advance this pipeline by enhancing dense point-wise interaction and geometric contextual aggregation.These methods adopt dual-branch encoding, cross-modal fusion and keypoint voting, and they show strong results on YCB-Video and LINEMOD. They are also competitive with leading RGB-only methods such as GDRNPP in several settings [32]. However, a fundamental limitation persists in the image branch. Despite these advancements, a critical research gap remains at the fundamental level of feature extraction. Most existing methods still rely on standard convolutions with fixed, square receptive fields. Since changes in 3D pose induce large variations in the 2D projection’s shape and scale, this rigid sampling grid fundamentally fails to align with the true appearance geometry. This geometric mismatch injects bias at the very beginning of the pipeline, meaning that even the most powerful subsequent fusion mechanisms are essentially constrained to compensating for a distorted representation rather than resolving the root cause [33,34,35].

### 2.2. Render and Compare Methods and Cross-Modal Transformer Fusion

To further push the performance boundaries, two main lines centered on enhancing backend processing capabilities have emerged. The first line of work strengthens the later processing stage through render and compare. DeepIM formulates pose refinement as image registration; it renders the current estimate and regresses a residual in an iterative loop to approach the true pose [36,37]. CosyPose extends this idea to multi-object and multi-view settings and enforces geometric consistency to improve global accuracy, while other works have explored novel rendering methods like NeRFs for the same purpose [13,38,39]. MegaPose, along with other strong refiners like GenFlow and SCARF, advances the render-and-compare paradigm, for example, by generalizing to novel objects after large-scale synthetic pre-training [12,40]. This family explicitly optimizes visible surface consistency and often achieves high scores on the occlusion robust metric VSD [41,42]. However, their inference requires multiple rendering and comparison steps, and the cost in computation and latency is high [39,41]. Furthermore, their pipeline relies on high-quality CAD models, accurate camera calibration, and agreement between rendered and real scenes, which limits its deployment under real-time and low-power constraints.

The second line of work strengthens fusion with cross-modal Transformers and heavy attention. Methods like DFTr and CATRE apply cross-attention between image and point cloud features to model long-range dependencies and align cross-modal evidence [14,43]. MaskedFusion, ZebraPose, and Fusion-Flow improve fusion under challenging conditions like occlusion or lack of texture through various mask-based or correspondence-based attention mechanisms [44,45,46]. Deformable Fusion Transformer further increases efficiency by learning sparse correspondences through deformable attention [47,48]. These methods leverage global dependency modeling and deliver gains on ADD-S and VSD. However, this performance often comes with increased parameter counts, memory footprints, and inference times [47,48]. Crucially, a limitation persists in the feature extraction stage: these fusion modules typically assume that input image features are geometrically aligned. When the receptive fields in early convolutions fail to match the projected morphology of the object, the subsequent fusion operates on biased features, limiting its ability to resolve the underlying geometric error [14,49,50].

### 2.3. Uncertainty Modeling and Fused Feature Quality

A fundamental challenge in 6D pose estimation is handling the inherent ambiguity arising from object symmetries, heavy occlusion, or textureless surfaces. Standard deterministic regression with losses like L1 or L2 struggles in these scenarios, as penalizing deviations from a single ground truth can force the model to predict a physically implausible average pose. This is a known pitfall when using Euclidean losses for ambiguous targets. The challenge of ambiguity has motivated a significant line of research into uncertainty modeling. Early works learned per-pixel uncertainty for coordinate regression [15], while later methods made the entire pose hypothesis-and-selection process differentiable [51,52]. More recently, the field has advanced towards estimating full probabilistic distributions over the pose space, using techniques like matrix Fisher distributions or implicit energy-based models [53]. However, despite these advances in modeling uncertainty, its application at inference time often remains limited. In most methods, the predicted variance is primarily used as a final confidence score or as an adaptive weight during the training process [54]. The regression head itself still typically outputs a single, deterministic pose, thereby failing to fully embrace a probabilistic approach to handle ambiguity. This overlooks the opportunity to use the predicted distribution to directly represent the multi-modal nature of ambiguous scenarios (e.g., symmetric objects), leaving a gap in developing regression mechanisms that are inherently more robust to such challenges.

In parallel with the challenges in the final pose calculation, the robustness of the entire pipeline is equally contingent on the quality of features at intermediate stages. For example, architectures centered on keypoint voting [8,11] depend on high-quality multi-modal features to generate accurate votes. However, common fusion operations like feature concatenation or summation implicitly treat information from both RGB and depth streams as equally reliable at all spatial locations [55]. This assumption is often invalid, as the fusion process can introduce significant noise and redundancy where artifacts from one modality may corrupt the representation of the other [1]. While complex backend modules like cross-modal Transformers perform some implicit feature re-weighting, they do so at a substantial computational cost [43,47,56]. While backend modules such as cross-modal Transformers perform implicit feature re-weighting, they often require high computational resources [43,47,56]. This indicates an unaddressed gap: the lack of a lightweight fusion attention mechanism capable of explicitly denoising and refining features after cross-modal interaction. Addressing this gap ensures that subsequent network layers process a cleaner representation, avoiding the computational overhead associated with heavy backend solutions.

## 3. Methodology

This section provides a detailed description of the proposed Pose-Perceptive Fusion Network (PPF-Net). When a three-dimensional object is projected onto a two-dimensional image plane, its visual morphology can vary under different poses, leading to scale differences. The objective of this network is to address shape and scale discrepancies in 6D object pose estimation caused by pose variations. To tackle this, PPF-Net extends the classic FFB6D framework and introduces an adaptive convolution operation. This operation adapts the receptive-field aspect ratio and the number of sampling points, improving feature extraction for objects with diverse shapes and scales. The contents of this section are organized as follows: First, the overall design and data processing pipeline of PPF-Net are introduced. Subsequently, the core Pose-Perceptive Convolution (PPC) module is elaborated upon. Finally, the Lightweight Fusion Attention module (LFA) and the probabilistic pose regression method incorporated in this study are described.

### 3.1. Overall Architecture of PPF-Net

**Figure 2:**
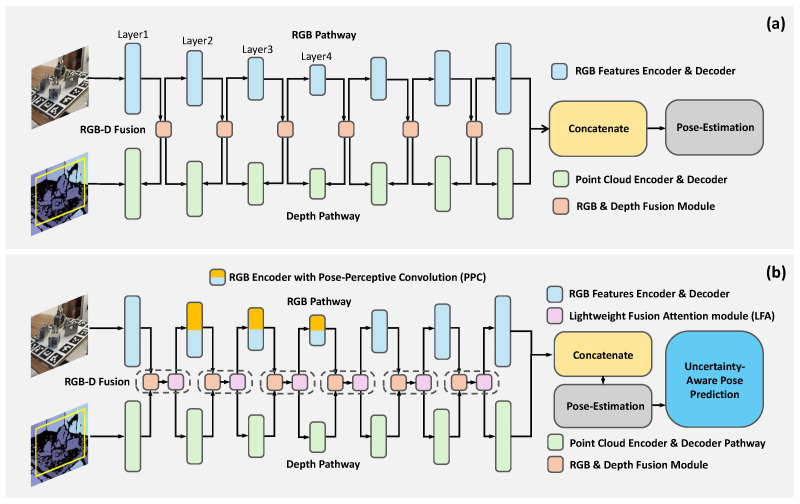
Comparison between the FFB6D (**a**) and the proposed PPF-Net (**b**). PPF-Net introduces several key enhancements, including the Pose-Perceptive Convolution (PPC) within the RGB encoder, a Lightweight Fusion Attention (LFA) module for feature refinement, and a probabilistic pose regression head for uncertainty-aware predictions. These additions improve the network’s robustness to varying object shapes and scales, enhancing both appearance and geometric representation for more accurate 6D pose estimation.

The proposed PPF-Net follows the cross-modal fusion strategy of FFB6D, which, as shown in Figure 2a, employs a dual-branch encoder-decoder with Bidirectional Fusion Modules to facilitate iterative information exchange between color and geometry modalities at each level of the feature encoding process. The overall design of PPF-Net is illustrated in Figure 2b. PPF-Net introduces a Pose-Perceptive Convolution module, a Lightweight Fusion Attention module, and a probabilistic regression head. These additions are designed to address the challenges in traditional 6D object pose estimation, specifically the issues of varying object shapes and scales, information redundancy in fused features, and inherent pose ambiguities.

**Figure 3:**
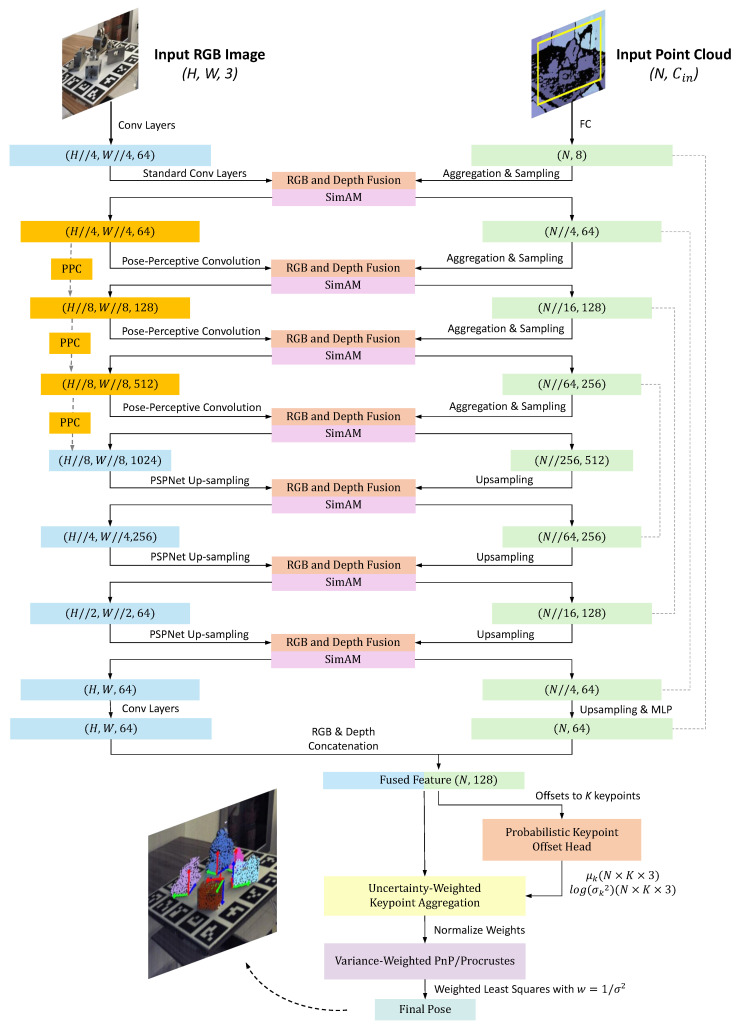
The detailed architecture of proposed PPF-Net. The RGB branch employs the Pose-Perceptive Convolution in deep layers to adapt the receptive field aspect ratio and sampling density, aligning features with object shape and scale. During encoding, RGB and point-cloud features are fused bidirectionally at each scale and a SimAM module [61] is inserted right after each fusion for denoising and enhancement. The point cloud branch learns multi-scale features through set aggregation and sampling, then upsamples in the decoder and concatenates with encoder features. The fused per-point features are fed to a probabilistic keypoint offset head that outputs a mean and a log-variance for *K* keypoints with tensor size N×K×3. Offsets and uncertainties are used in an uncertainty-weighted keypoint aggregation that normalizes weights and yields keypoint coordinates. The aggregated keypoints are finally solved by variance-weighted PnP or Procrustes using weighted least squares with $w=1/\sigma^{2}$, producing the final 6D pose.

The detailed architecture of the proposed PPF-Net is illustrated in Figure 3. The data processing pipeline begins with an RGB image and a corresponding point cloud as inputs. The RGB image is fed into a newly designed Pose-Perceptive Encoder, which replaces the standard convolutional layers in the deeper stages of the original backbone with our proposed Pose-Perceptive Convolution (PPC) modules. This modification allows the network to adaptively extract features that align with the specific shape and scale of the object’s 2D projection. Concurrently, the point cloud is processed by the RandLA-Net network [57], which serves as the hierarchical point feature extractor.

At each corresponding scale within the encoder, the features from the Pose-Perceptive Encoder and the point feature extractor are passed through the Bidirectional Fusion Modules. This step ensures deep integration of appearance and geometric information. To further refine the fused features, a Lightweight Fusion Attention (LFA) module is applied immediately after each fusion operation. The LFA module selectively emphasizes informative features while suppressing potential noise or redundancy introduced during the fusion process.

In the decoder stage, feature propagation modules are utilized to progressively upsample the point features to their original resolution. These upsampled features are combined with multi-scale features from the encoder via skip connections, preserving both high-level semantic context and fine-grained spatial details. Finally, instead of regressing a deterministic pose, the per-point features are passed to a probabilistic prediction head. This head estimates the mean and variance for the offsets to predefined 3D keypoints, enabling the model to quantify the uncertainty associated with its predictions. The final 6D pose is then computed from the estimated keypoint locations.

### 3.2. Pose-Perceptive Convolution for Adaptive Feature Extraction

A primary challenge in RGB-D based 6D object pose estimation is the significant geometric variation of objects as projected onto the 2D image plane; this variation manifests as substantial differences in shape and scale, not only between different object instances but also for the same object viewed from varying perspectives [8,45]. Within the RGB feature extraction branch, standard convolutional networks employ kernels with a fixed, square receptive field. As feature maps progress to deeper layers, this rigid sampling geometry creates a fundamental mismatch with the diverse and non-uniform shapes of the objects being analyzed [34,58]. This mismatch leads to the extraction of low-quality or incomplete 2D features, since the fusion process relies on high-quality 2D appearance features to provide a semantic context for the 3D point cloud, inaccurate 2D feature representation directly compromises the quality of the fused multi-modal features [9,10], ultimately degrading the accuracy of the final pose estimation.

**Figure 4:**
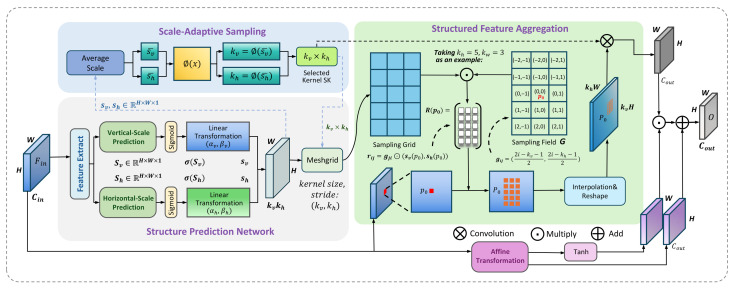
Overview of the Pose-Perceptive Convolution architecture. This module consists of three main parts. The first part handles the learning of the convolutional kernel’s height and width, which is referred to as the Structure Prediction Network (SPN). The second part, Scale-Adaptive Sampling, focuses on dynamically adjusting the sampling density. The final part demonstrates the process of feature aggregation guided by a structured sampling field, using a specific grid position $p_{0}$ as an example.

Deformable Convolution (DCN) [58,59] was introduced to mitigate this issue by learning offsets to adaptively adjust the sampling positions of the kernel. While it provides flexibility in sampling location, two critical limitations persist. First, the unstructured nature of its sampling field, which lacks geometric priors, can increase the risk of focusing on irrelevant contextual information instead of the object’s intrinsic structure [35]. More importantly, the number of sampling points remains fixed, preventing the kernel from adjusting its sampling density in response to the object’s perceived scale [33,60]. Therefore, to address these specific limitations, we propose the Pose-Perceptive Convolution (PPC), as shown in Figure 4. PPC is explicitly designed to perceive the geometric structure of an object’s projection by dynamically learning two critical properties: the principal axis scales, which determine the aspect ratio, and a corresponding sampling density.

#### 3.2.1. Dynamic Structure Perception

As shown in Figure 4, the core mechanism of PPC is its ability to dynamically perceive the geometric structure of objects at each spatial location in the feature map. This is achieved through a submodule named the Structure Prediction Network (SPN). The SPN takes the input feature map $F_{in}\in R^{H\times W\times C_{in}}$ and predicts two corresponding scale maps, the vertical scale map $S_{v}$ and the horizontal scale map $S_{h}$. This process can be formulated as follows:

(1) $$S_{v},S_{h}=\mathrm{SPN}\left(F_{in};\theta_{spn}\right)$$

where $\theta_{spn}$ represents the learnable parameters of the SPN. The SPN consists of a shared feature encoder followed by two separate prediction heads. Each head is responsible for predicting one of the scale maps, $S_{v},S_{h}\in R^{H\times W\times 1}$. To ensure the predicted scales are bounded and stable for training, the final activation function in each prediction head is a Sigmoid function, which maps the outputs to the range (0,1).

However, these normalized values only represent relative scales and cannot be used directly as the dimensions of the receptive field. Therefore, a linear transformation is applied to remap these values into a predefined, meaningful range. The final principal axis scales, denoted as $s_{v}$ and $s_{h}$ for a single location, are computed as

(2) $$s_{v}=\alpha_{v}\cdot \sigma \left(S_{v}\right)+\beta_{v}$$

(3) $$s_{h}=\alpha_{h}\cdot \sigma \left(S_{h}\right)+\beta_{h}$$

where σ(·) is the Sigmoid function of SPN. The parameters $\alpha_{v},\alpha_{h}$ are scaling factors and $\beta_{v},\beta_{h}$ are bias terms, which are hyperparameters that define the operational range of the kernel’s dimensions. Specifically, the vertical dimension of the receptive field is constrained within $\left(\beta_{v},\alpha_{v}+\beta_{v}\right)$, and the horizontal dimension is constrained within $\left(\beta_{h},\alpha_{h}+\beta_{h}\right)$. These dynamically computed scales, $s_{v}$ and $s_{h}$, provide the geometric basis for the subsequent steps of sampling and feature aggregation.

The dynamic structure perception process described above is summarized in Algorithm 1. It enables the network to learn a location-specific aspect ratio for its receptive field, directly addressing the problem of feature mismatch caused by fixed, square kernels of standard convolutions and thereby allowing the network to capture more accurate and complete geometric information from the RGB image.
**Algorithm 1** Dynamic Structure Perception 1:**Input:** Input feature map $F_{\mathrm{in}}\in R^{H\times W\times C_{in}}$ 2:**Hyperparameters:** Scaling factors $\alpha_{v},\alpha_{h}$; Bias terms $\beta_{v},\beta_{h}$ 3:**Output:** Principal axis scale maps $S_{v}^{\prime },S_{h}^{\prime }$ 4:$S_{v},S_{h}\leftarrow \mathrm{SPN}\left(F_{\mathrm{in}}\right)$       ▹ Outputs are in range (0, 1) due to internal Sigmoid 5:$S_{v}^{\prime }\leftarrow \alpha_{v}\cdot S_{v}+\beta_{v}$    ▹ Remap normalized scales to a meaningful operational range 6:$S_{h}^{\prime }\leftarrow \alpha_{h}\cdot S_{h}+\beta_{h}$ 7:**return **$S_{v}^{\prime },S_{h}^{\prime }$

#### 3.2.2. Scale-Adaptive Sampling

In addition to adapting the aspect ratio of the receptive field, PPC also dynamically adjusts its sampling density. The optimal sampling density for feature extraction varies with network depth: early layers require higher-density sampling to capture fine-grained details, whereas deeper layers benefit from sparser sampling to grasp holistic semantic structures [33,62]. However, standard convolutions are inherently limited in this regard. They employ a fixed sampling density throughout the network, which fails to adapt to these varying scales and levels of abstraction, consequently hindering effective feature capture [58,63].

To address this, PPC introduces a scale-adaptive sampling mechanism. Instead of using a fixed number of sampling points, the sampling density is determined based on the globally aggregated principal axis scales learned in the previous step. Specifically, the average vertical and horizontal scales, denoted as ${\bar{s}}_{v}$ and ${\bar{s}}_{h}$ are first computed across all spatial of the scale maps $S_{v}$ and $S_{h}$.

(4) $${\bar{s}}_{v}=\frac{1}{H\times W}\sum_{i=1}^{H}\sum_{j=1}^{W}s_{v}^{\left(i,j\right)},\;{\bar{s}}_{h}=\frac{1}{H\times W}\sum_{i=1}^{H}\sum_{j=1}^{W}s_{h}^{\left(i,j\right)}$$

where $s_{v}^{\left(i,j\right)}$ and $s_{h}^{\left(i,j\right)}$ are the principal axis scales at location (i,j). These average scales reflect the overall geometric properties of the features at the current network layer. Based on these values, the number of sampling points in the vertical and horizontal directions, $k_{v}$ and $k_{h}$, are determined by a mapping function ϕ(·):

(5) $$k_{v}=\phi \left({\bar{s}}_{v}\right),\;k_{h}=\phi \left({\bar{s}}_{h}\right)$$

The function ϕ(x) is designed to convert a continuous scale value into an odd integer, ensuring that the sampling grid has a distinct center. It is defined as follows:

(6) ϕ(x)=⌊x⌋−I(⌊x⌋mod2=0)

where ⌊·⌋ is the floor function and I(·) is the Iverson bracket, which returns 1 if the condition inside is true, and 0, otherwise. This formulation ensures that if the floor of the average scale is an even number, the function selects the next smaller odd number.

The scale-adaptive sampling process described above is summarized in Algorithm 2. This mechanism enables each PPC layer to autonomously select an appropriate sampling density. This allows the network to use a denser grid for detailed feature extraction in shallow layers where average learned scales are small, and a sparser, more computationally efficient grid in deeper layers where the network focuses on larger contextual structures.
**Algorithm 2** Scale-Adaptive Sampling 1:**Input:** Principal axis scale maps $S_{v}^{\prime },S_{h}^{\prime }$ from Algorithm 1 2:**Output:** Number of sampling points $k_{v},k_{h}$ 3:**function **ϕ (*x*)          ▹ Define the mapping function to an odd integer 4:    **return **⌊x⌋−I(⌊x⌋mod2=0) 5:**end function** 6:${\bar{s}}_{v}\leftarrow \mathrm{mean}\left(S_{v}^{\prime }\right)$      ▹ Compute average scales across the spatial dimensions 7:${\bar{s}}_{h}\leftarrow \mathrm{mean}\left(S_{h}^{\prime }\right)$ 8:$k_{v}\leftarrow \phi \left({\bar{s}}_{v}\right)$              ▹ Determine the number of sampling points 9:$k_{h}\leftarrow \phi \left({\bar{s}}_{h}\right)$10:**return **$k_{v},k_{h}$

#### 3.2.3. Structured Feature Aggregation

With the principal axis scales ($s_{v},s_{h}$) and the sampling density ($k_{v}\times k_{h}$) determined, PPC generates a structured sampling field to guide the feature aggregation process. Unlike standard convolution, which samples on a rigid integer grid, PPC defines a sampling grid G of size $k_{v}\times k_{h}$ in a normalized coordinate system. An element $g_{ij}\in G$ at the *i*-th row and *j*-th column is defined as

(7) $$g_{ij}=\left(\frac{2i-k_{v}-1}{2},\frac{2j-k_{h}-1}{2}\right)$$

where $1\leq i\leq k_{v}$ and $1\leq j\leq k_{h}$. This normalized grid is then scaled by the locally predicted principal axis scales ($s_{v},s_{h}$) at each spatial position $p_{0}$ on the input feature map $F_{in}$. This creates a location-specific offset matrix $R(p_{0})$, which constitutes the structured sampling field. An offset $r_{ij}\in R\left(p_{0}\right)$ is computed by element-wise multiplication:

(8) $$r_{ij}\left(p_{0}\right)=g_{ij}⊙\left(s_{v}\left(p_{0}\right),s_{h}\left(p_{0}\right)\right)=\left(\frac{2i-k_{v}-1}{2}s_{v}\left(p_{0}\right),\frac{2j-k_{h}-1}{2}s_{h}\left(p_{0}\right)\right)$$

The sampling points defined by $R(p_{0})$ are not restricted to integer coordinates. Therefore, bilinear interpolation is employed to sample the feature values from $F_{in}$ at the new locations $p_{0}+r_{ij}$. Let F(p) denote the feature vector at a generic position *p*. The final feature aggregation at position $p_{0}$ for the output feature map O is then a weighted sum over the structured sampling field:

(9) $$O\left(p_{0}\right)=\sum_{r_{ij}\in R\left(p_{0}\right)}W_{ij}\cdot F\left(p_{0}+r_{ij}\right)$$

where $W_{ij}$ represents the learnable weights of the convolutional kernel corresponding to the grid position (i,j).

The structured feature aggregation process described above is summarized in Algorithm 3. On one hand, it is highly parameter-efficient, as the entire sampling field is derived from only two learned principal axis scale values, rather than learning independent offsets for each sampling point. On the other hand, the sampling points of PPC maintain a grid structure that is scaled anisotropically, which encourages the network to learn features related to an object’s intrinsic shape rather than arbitrary spatial correlations, thereby providing a better inductive bias for perception tasks.
**Algorithm 3** Structured Feature Aggregation 1:**Input:** Input feature map $F_{\mathrm{in}}$; Principal axis scale maps $S_{v}^{\prime },S_{h}^{\prime }$; Number of sampling points $k_{v},k_{h}$; Learnable kernel weights W 2:**Output:** Output feature map O 3:**for **i=1 to $k_{v}$** do**              ▹ Define the normalized sampling grid 4:    **for** j=1 to $k_{h}$ **do** 5:        $G_{ij}\leftarrow \left(\frac{2i-k_{v}-1}{2},\frac{2j-k_{h}-1}{2}\right)$ 6:    **end for** 7:**end for** 8:        ▹ Perform structured sampling and aggregation for each position 9:**for all** spatial positions $p_{0}$ in $F_{\mathrm{in}}$ **do**10:    $O(p_{0})\leftarrow 0$11:    Get local scales $s_{v}\left(p_{0}\right),s_{h}\left(p_{0}\right)$ from $S_{v}^{\prime },S_{h}^{\prime }$12:    **for** i=1 to $k_{v}$ **do**13:        **for** j=1 to $k_{h}$ **do**14:           $r_{ij}\leftarrow G_{ij}⊙\left(s_{v}\left(p_{0}\right),s_{h}\left(p_{0}\right)\right)$          ▹ Compute local offset15:                 ▹ Sample features using bilinear interpolation16:           $F_{sampled}\leftarrow \mathrm{BilinearInterpolation}\left(F_{\mathrm{in}},p_{0}+r_{ij}\right)$17:           $O\left(p_{0}\right)\leftarrow O\left(p_{0}\right)+W_{ij}\cdot F_{sampled}$     ▹ Aggregate weighted features18:        **end for**19:    **end for**20:**end for**21:**return **O

#### 3.2.4. Comparison with Deformable Convolutions

While both Pose-Perceptive Convolution (PPC) and Deformable Convolution (DCN) [58] fall under the category of adaptive convolution, they differ fundamentally in their modeling objective, deformation structure, and efficiency.

**Figure 5:**
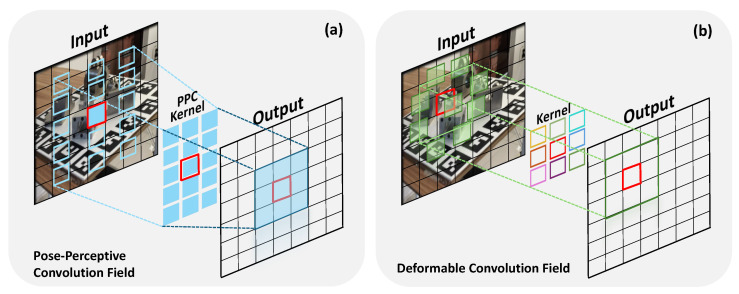
Comparison of sampling fields between (**a**) the proposed Pose-Perceptive Convolution (PPC) and (**b**) Deformable Convolution (DCN). DCN learns free-form, unstructured offsets for each sampling point, whereas PPC learns a structured, anisotropic field that aligns with the object’s overall projected geometry.

First, as illustrated in Figure 5, the core modeling objectives diverge. DCN allows each sampling point to move independently by learning unstructured, per-point offsets [58]. In contrast, PPC is designed to align the overall shape of the receptive field with the 2D projected geometry of the object, representing a structured geometric alignment process. PPC achieves this through anisotropic deformation by learning two macroscopic principal-axis scales, rather than microscopic, independent point displacements.

Specifically, in PPC, the sampling location is determined jointly by a shared normalized grid point $g_{ij}$ and two shared scales, $s_{v}$ and $s_{h}$, learned at the center location $p_{0}$:

(10) $$p_{ppc}=p_{0}+g_{ij}⊙\left(s_{v}\left(p_{0}\right),s_{h}\left(p_{0}\right)\right)$$

This formulation highlights that unlike DCN, which assigns independent degrees of freedom to each sampling point, PPC constrains all sampling points by two shared scale parameters, thereby enforcing a structured topological relationship.

Table 1 summarizes the distinctions between PPC and DCN. In our setting, PPC focuses on geometry-aligned receptive-field shaping using only two scale parameters per location, whereas DCN introduces $2k^{2}$ offset degrees of freedom. This design makes PPC more targeted to geometric alignment in pose estimation while remaining parameter-efficient.

### 3.3. Fusion Denoising and Uncertainty-Aware Regression

The integration of the PPC module significantly enhances the network’s ability to extract geometrically-aligned features from the RGB input. However, two challenges inherent to the FFB6D method remain unaddressed: the potential for information redundancy in the fused features [1], and the deterministic nature of the final pose regression, which struggles with inherent ambiguities common in pose estimation. To address these issues, this study further introduces two auxiliary enhancements: a Lightweight Fusion Attention (LFA) module for feature refinement and a probabilistic regression method for uncertainty-aware prediction.

#### 3.3.1. Feature Denoising with Lightweight Fusion Attention

**Figure 6:**
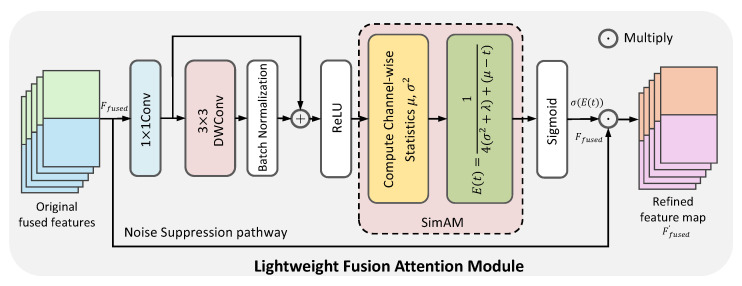
The architecture of the Lightweight Fusion Attention (LFA) module. The module consists of a feature enhancement pathway followed by a SimAM-based attention block. It takes the fused features as input, estimates 3D attention weights based on neuronal energy, and applies these weights to the original input to output a denoised feature representation.

The Bidirectional Fusion Modules in FFB6D effectively combine appearance and geometric information [10]. However, in challenging scenarios involving heavy occlusion or specular reflections, this direct fusion may introduce noisy or invalid features from one modality into the other, such as RGB features from occluded areas or erroneous depth information from reflective surfaces. This feature contamination can dilute the quality of the fused representation and compromise the final pose estimation robustness. To suppress this noise and refine the fused features, the Lightweight Fusion Attention (LFA) module is introduced, as shown in Figure 6.

The LFA module incorporates a parameter-free attention mechanism adapted from SimAM [61] to evaluate feature importance. Unlike complex attention modules that require heavy learnable parameters, SimAM efficiently estimates 3D attention weights by calculating an energy function based on the statistical distinction between a target neuron and its neighbors. This energy-based weighting is integrated into the fusion pipeline to selectively emphasize informative neurons while suppressing redundant ones. The refined feature map $F_{fused}^{\prime }$ is obtained by an element-wise multiplication of the original fused features $F_{fused}$ with the generated energy-based attention map E(t), scaled by a Sigmoid function:

(11) $$F_{fused}^{\prime }=F_{fused}⊙\sigma \left(E\left(t\right)\right)$$

The feature denoising process is outlined in Algorithm 4. The LFA module first passes the features through a Conv-DWConv-Residual pathway and then applies the SimAM-based weighting. This module is applied immediately after each Bidirectional Fusion Module. This strategic placement ensures that feature refinement occurs at every stage of the cross-modal interaction, allowing for the timely suppression of noise and enhancement of salient features, which leads to a cleaner and more potent feature representation for subsequent layers.
**Algorithm 4** Lightweight Fusion Attention (LFA) 1:**Input:** Fused feature map $F_{fused}$ 2:**Hyperparameter:** Regularization term λ 3:**Output:** Refined feature map $F_{fused}^{\prime }$ 4:               ▹ Process through the feature enhancement pathway 5:$F_{enhanced}\leftarrow \mathrm{ReLU}\left(\mathrm{BN}\left(\mathrm{DWConv}\left(\mathrm{Conv}\left(F_{fused}\right)\right)\right)+F_{fused}\right)$ 6:          ▹ Generate attention map using SimAM principles [61] 7:Compute energy map *E* based on channel-wise statistics of $F_{enhanced}$ 8:A←σ(E)           ▹ Apply attention weights to the original features 9:$F_{fused}^{\prime }\leftarrow F_{fused}⊙A$10:**return **$F_{fused}^{\prime }$

#### 3.3.2. Probabilistic Regression for Uncertainty-Aware Pose Prediction

Many pose estimation methods rely on deterministic regression, such as L1 or L2 loss. In cases involving object symmetry or heavy occlusion, this approach struggles with inherent ambiguities. Forcing a single deterministic prediction can lead to suboptimal training, often causing the model to converge to a physically implausible average pose, as noted in prior work [64].

To address this, the deterministic prediction head is replaced with a probabilistic one. Instead of predicting a single value, the network learns a probability distribution. Specifically, for each keypoint offset, the network predicts a mean μ and a variance $\sigma^{2}$, representing the predicted value and the associated uncertainty, respectively.

The heteroscedastic aleatoric uncertainty loss [54] is employed to train the network. For a ground truth offset *y* and a predicted distribution parameterized by μ(x) and $\sigma^{2}\left(x\right)$, the Gaussian negative log-likelihood (NLL) loss is formulated as

(12) $$L_{\mathrm{NLL}}\left(y,\mu \left(x\right),\sigma^{2}(x)\right)=\frac{||y-{\mu \left(x\right)||}^{2}}{2\sigma^{2}\left(x\right)}+\frac{1}{2}\log\left(\sigma^{2}\left(x\right)\right)$$

This loss function naturally down-weights the penalty for samples with high predicted uncertainty, allowing the model to be robust to noisy data or ambiguous poses without requiring explicit outlier rejection steps during training. The overall procedure is summarized in Algorithm 5.
**Algorithm 5** Probabilistic Regression for Uncertainty-Aware Pose Prediction 1:**Input:** Per-point features $F_{point}$; Ground truth keypoint offsets $Y_{gt}$ 2:**Network:** Probabilistic Prediction Head (a Multi-Layer Perceptron, MLP) 3:**Output:** Predicted mean offsets μ; Predicted log variances v (where $\sigma^{2}=\exp\left(v\right)$)**Training Phase:** 4:       ▹ The MLP predicts a mean and a log variance for each keypoint offset 5:$\mu ,v\leftarrow \mathrm{MLP}(F_{point})$      ▹ Convert log variance to variance for the loss calculation 6:$\sigma^{2}\leftarrow \exp\left(v\right)$                  ▹ Ensures variance is always positive 7:           ▹ Compute the Gaussian Negative Log-Likelihood (NLL) Loss 8:$L_{\mathrm{data}}\leftarrow \frac{\left|\right|Y_{gt}-\mu {\left|\right|}^{2}}{2\sigma^{2}}$        ▹ Data-dependent error, down-weighted by variance  9:$L_{\mathrm{reg}}\leftarrow \frac{1}{2}\log\left(\sigma^{2}\right)$          ▹ Regularization term, penalizes high uncertainty 10:$L_{\mathrm{NLL}}\leftarrow L_{\mathrm{data}}+L_{\mathrm{reg}}$        ▹ Update network parameters by minimizing $L_{\mathrm{NLL}}$**Inference Phase:**11:   ▹ The network outputs the mean as the prediction and variance as confidence12:$\mu_{pred},v_{pred}\leftarrow \mathrm{MLP}\left(F_{point}\right)$13:$\sigma_{pred}^{2}\leftarrow \exp\left(v_{pred}\right)$14:  ▹$\sigma_{pred}^{2}$ is used as a reliability signal for downstream tasks (e.g., weighted ICP).15:**return **$\mu_{pred},\sigma_{pred}^{2}$       ▹$\mu_{pred}$ is used as the keypoint offset prediction.

## 4. Experimental Results and Analyses

This section evaluates the proposed PPF-Net on multiple benchmark datasets under official protocols. The experimental settings, including datasets, evaluation metrics, and implementation details, are introduced first. Subsequently, the main quantitative comparisons are reported, followed by ablation studies and further analyses to examine the contribution of each component.

### 4.1. Benchmark Datasets

The proposed method is evaluated on four widely used and challenging benchmark datasets to comprehensively validate its performance and robustness.

**MP6D** [65] dataset consists of 20 industrial metal components captured in environments with severe occlusion and significant illumination changes. The objects are characterized by textureless and highly reflective surfaces, complex geometries, and symmetrical properties. This dataset is therefore particularly suited for evaluating the model’s robustness under challenging industrial conditions. The experiments follow the official training and testing split provided by the benchmark.

**YCB-Video** [19] dataset is a standard benchmark for 6D pose estimation. It comprises 21 common household objects captured in cluttered scenes with varying levels of occlusion. It serves as the primary benchmark for evaluating the overall performance of the method and for comparison with other state-of-the-art methods. The standard data processing and train/test split protocols established by prior works are adhered to.

**LINEMOD** [2] is a classic benchmark dataset featuring 13 objects with limited or no texture. Success on this dataset requires the model to rely heavily on geometric shape information for pose determination. Following common practice, the training set is augmented with synthetic images, and the established training and testing splits are used.

**Occlusion-LINEMOD** [16] dataset is a highly challenging subset derived from LINEMOD. Each scene contains multiple objects with severe inter-object occlusion. This dataset is specifically used to test the robustness of the method under extreme occlusion scenarios, which is a key focus of the proposed enhancements.

### 4.2. Evaluation Metrics

The performance of the method is assessed using three standard 6D pose evaluation metrics established in the field: the Average Distance of Model Points (ADD) [2], the Average Closest Point Distance (ADD-S) [2], and the Visible Surface Discrepancy (VSD) [5].

The **ADD** metric is utilized for asymmetric objects. It computes the mean distance between the set of 3D model vertices transformed by the ground truth pose and the estimated pose. For an object model M with *N* vertices, it is formulated as follows [2]:

(13) $$L_{\mathrm{ADD}}=\frac{1}{N}\sum_{p\in M}∥\left(Rp+t\right)-\left(\hat{R}p+\hat{t}\right)∥$$

where R,t are the ground truth pose and $\hat{R},\hat{t}$ are the estimated pose.

The **ADD-S** metric is designed for symmetric objects to handle pose ambiguity. It computes the mean distance between each vertex of the ground truth model and its closest corresponding vertex in the estimated model [2], formulated as

(14) $$L_{\mathrm{ADD}-S}=\frac{1}{N}\sum_{p_{1}\in M}\min_{p_{2}\in M}∥\left(Rp_{1}+t\right)-\left(\hat{R}p_{2}+\hat{t}\right)∥$$

The **VSD** metric is a rendering-based measure that is invariant to object symmetries. It evaluates the error by comparing the discrepancy between the rendered visible depth surfaces of the object from the estimated and ground truth poses. This metric is robust to occlusion as it only considers visible surface areas [5,66].

The specific metrics used for each dataset adhere to their respective official evaluation conventions:

1. On the **YCB-Video** dataset, we report the Area Under the Curve (AUC) scores for the **ADD-S** and **ADD(-S)** metrics, following the protocol defined in PoseCNN [19]. ADD(-S) is a hybrid metric that applies ADD for asymmetric objects and ADD-S for symmetric ones.
2. On the **MP6D** dataset, the AUC scores for the **ADD-S** and **VSD** metrics are reported to comprehensively evaluate both geometric and visible surface alignment accuracy in cluttered scenes, as recommended in [65].
3. On the **LINEMOD** and **Occlusion-LINEMOD** datasets, the accuracy at **ADD(-S) < 0.1d** is reported [16]. This metric calculates the percentage of samples where the prediction error is less than 10% of the object’s diameter.

Furthermore, to assess the computational efficiency and deployment feasibility, we report the following metrics:

1. **Model Complexity:** Measured by the number of learnable parameters (Params) and Floating Point Operations (FLOPs) for a single forward pass.
2. **Inference Speed:** Evaluated using end-to-end Latency (ms), measured on a single GPU with a batch size of 1.
3. **Resource Usage:** Monitored via Peak Memory consumption during inference to gauge the hardware requirements.

### 4.3. Implementation Details

To ensure a fair and direct comparison, the experimental setup strictly adheres to the protocol of the FFB6D baseline. Input data consists of 480 × 640 resolution RGB images and point clouds comprising 12,288 points sampled from the depth map. For the geometric branch, feature extraction from the point cloud is performed by the RandLA-Net backbone. The appearance branch utilizes an ImageNet-pretrained ResNet-34 as the encoder, complemented by a four-level PSPNet for feature decoding.

The Pose-Perceptive Convolution (PPC) modules are strategically integrated into the RGB encoder, replacing the standard 3×3 convolutions in layer 2, layer 3, and layer 4 of the ResNet-34 backbone. The initial layer 1 is kept unchanged to maintain stable extraction of low-level features. For cross-modality fusion, the bidirectional design from FFB6D is retained. A Lightweight Fusion Attention (LFA) module, based on SimAM, is inserted immediately after each fusion block to refine the fused feature representation. Finally, the deterministic regression head is replaced with a probabilistic one, which is trained using the Gaussian negative log-likelihood loss.

For training, we strictly adhere to the official data splits and evaluation protocols used by the baseline [10]. The model is optimized using the AdamW optimizer combined with a learning rate schedule. To enhance robustness, we implement specific data augmentation strategies, including random color jittering for RGB images and random perturbation with dropout for point clouds. All random seeds are fixed to ensure reproducibility. Additionally, the variance predicted by the probabilistic head serves as a reliability weight for downstream refinement tasks, including weighted ICP. All quantitative results reported in the subsequent sections exclude iterative refinement steps unless explicitly stated.

### 4.4. Evaluation on Four Benchmark Datasets

To ensure a rigorous and fair evaluation, the comparisons are organized around the RGB-D fusion pipeline under the same single-view 6D pose setting and the official protocols of each benchmark. FFB6D is chosen as a widely used RGB-D fusion baseline to verify improvements over a canonical fusion architecture, and RCVPose is included as a strong RGB-D method that enhances the voting and geometric consistency stage. Furthermore, RDPN6D and CMAGCA are incorporated as recent RGB-D approaches that strengthen cross-modal interaction, reflecting current progress within the same pipeline family. In addition, several reference methods from different paradigms are reported to provide context rather than a strict like-for-like comparison: GDRNPP represents RGB-only pose estimation without depth input, while MegaPose and DFTr represent heavier backend designs based on render-and-compare or Transformer-based fusion. Unless explicitly marked with ICP, all results are reported without iterative refinement to isolate the contribution of the core network.

#### 4.4.1. Evaluation on the MP6D Dataset

Table 2 presents the quantitative results of PPF-Net on the MP6D dataset. The results show that PPF-Net improves over the baseline FFB6D by 5.0 points on the ADD-S AUC metric (91.3 vs. 86.3) and by 19.4 points on the VSD metric (79.9 vs. 60.5). Compared to recent RGB-D methods, PPF-Net remains competitive. In comparison to RCVPose, PPF-Net achieves higher ADD-S scores on 15 out of 20 objects and exceeds its VSD performance on all 20 objects. We further include two recent RGB-D approaches, RDPN6D and CMAGCA, to reflect recent progress in cross-modal interaction: RDPN6D reports only the overall average result on MP6D (ADD-S AUC of 95.9) without per-object breakdown and VSD scores, while CMAGCA reports an average ADD-S AUC of 92.9 but does not report VSD results, which prevents a strict two-metric comparison. When compared with MegaPose, PPF-Net is slightly higher on the average ADD-S metric (91.3 vs. 91.1), while its VSD score is lower (79.9 vs. 84.1). Furthermore, compared to DFTr, PPF-Net is close on the VSD metric (79.9 vs. 80.3), with a difference of 0.4 points.

It is noteworthy that the performance gains of recent advanced methods often heavily rely on introducing more complex modules to enhance the model’s representation capacity. For instance, MegaPose leverages a large-scale render-and-compare mechanism to improve generalization, while DFTr depends on computationally intensive Transformer blocks to capture global dependencies. In contrast, CMAGCA adopts a more lightweight attention design to strengthen cross-modal interaction without adopting a high-cost multi-head cross-attention architecture. In contrast to these approaches, the proposed PPF-Net does not follow the path of increasing model complexity, but rather returns to the fundamental problem of feature extraction. The focus is shifted to a widely overlooked issue: how to effectively handle the diverse morphologies and significant aspect ratio variations that objects exhibit on the RGB image. The improvement over FFB6D is obtained by replacing the standard convolutions in stages layer2 through layer4 of the ResNet, which allows the receptive field to adapt according to the object’s geometric structure.

This result suggests that existing works may have overemphasized enhancing the model’s abstract representation capacity, while underutilizing the foundational morphological features available in the RGB pathway. As demonstrated in the subsequent ablation study, the PPC module is the most critical factor for the performance gain.

Furthermore, the auxiliary enhancement modules play a crucial role in improving robustness. The SimAM-based attention module enhances feature selection in scenes with high reflectivity and occlusion, while the probabilistic regression head suppresses noise interference by predicting uncertainty and down-weighting votes from low-confidence regions. This is the key reason why the performance degradation of PPF-Net on the VSD metric is substantially smaller than that of previous methods.

#### 4.4.2. Evaluation on the YCB-Video Dataset

Table 3 presents the results on the YCB-Video dataset without iterative refinement. PPF-Net achieves an ADD-S score of 96.7, which is the same as DFTr (96.7), and higher than RCVPose (96.6) and GDRNPP (96.1). We further include two recent RGB-D approaches, CMAGCA and RDPN6D: CMAGCA reports 96.9 on ADD-S and 94.3 on ADD(-S) without ICP-based post-refinement, while RDPN6D reports 98.4 on ADD-S and 94.6 on ADD(-S) and states that its results are obtained without time-consuming post-processing. On the ADD(-S) metric, PPF-Net obtains 95.4, which is higher than DFTr (94.4), RCVPose (95.2), CMAGCA (94.3), and RDPN6D (94.6), while remaining below the render-and-compare based MegaPose (96.2). The performance gains can be attributed to the geometrically aligned receptive fields of PPC, which stabilize the ADD-S performance, while the SimAM-based attention and the probabilistic regression head are particularly crucial for the ADD(-S) improvement by suppressing noise and down-weighting unreliable evidence.

Table 4 shows the results with ICP refinement. The performance of PPF-Net is boosted to 97.8 on ADD-S and 96.0 on ADD(-S), representing an improvement of 1.1 and 0.6 points over the non-refined version, respectively. Notably, its refined ADD(-S) score is the highest among all non-render-and-compare methods in this table. CMAGCA and RDPN6D do not report ICP-refined results and are therefore marked as NR. This trend is consistent with the primary findings on the MP6D dataset, further validating the effectiveness of the proposed modules.

#### 4.4.3. Evaluation on the LINEMOD & Occlusion-LINEMOD Dataset

As shown in Table 5, performance on the standard LINEMOD dataset is close to saturation. PPF-Net yields a mean accuracy of 99.6%, demonstrating performance comparable to state-of-the-art methods ranging from DFTr (99.7%) and CMAGCA (99.8%) to MegaPose (99.6%). The minimal variance across methods indicates that the proposed frontend successfully captures the necessary features for objects with visible appearances.

In the more demanding scenario of Occlusion-LINEMOD (Table 6), the robustness of the model is rigorously tested. PPF-Net achieves a mean accuracy of 84.6%, showing a substantial improvement over the FFB6D baseline (66.2%) and RCVPose (79.2%). Furthermore, it compares favorably against recent RGB-D methods, outperforming RDPN6D (79.5%) and CMAGCA (73.6%). While the refinement-based MegaPose achieves the top rank with 85.0%, PPF-Net ranks second with a marginal gap of 0.4 points. At the object level, the method achieves the best performance on specific categories such as *eggbox* and maintains competitive stability across the dataset.

To assess computational efficiency, representative methods are profiled on a single NVIDIA A100 GPU with a batch size of 1, using RGB-D inputs resized to 480×640 with 12,288 points. Table 7 reports the number of parameters, FLOPs for a single pass (excluding ICP), peak memory, and end-to-end latency. Compared to the FFB6D baseline, the proposed PPF-Net introduces an increase of 2.7 M parameters and 7.3 ms in latency due to the additional offset prediction layers, resulting in an inference time of 35.7 ms. In comparison, the Transformer-based DFTr requires 132.4 M parameters and executes in 84.2 ms. For MegaPose, the standard inference configuration is profiled, which involves evaluating 50 coarse hypotheses followed by 5 render-and-compare refinement iterations. This mechanism results in 1450.5 G FLOPs and a latency of 465.0 ms, despite a parameter count of 42.6 M. These results indicate that PPF-Net improves geometric feature extraction with a lower computational burden compared to backend-intensive architectures.

### 4.5. Ablation Study

To investigate the sources of performance improvement, ablation studies are conducted on the key design choices of PPF-Net. Starting from the FFB6D baseline, the proposed modules are progressively integrated to assess their impact under a unified evaluation protocol. The effects of each component, both in isolation and in combination, are analyzed in the following subsections.

#### 4.5.1. Components Ablation on MP6D Dataset

A comprehensive ablation study is conducted on the MP6D dataset to validate the contribution of each proposed component in PPF-Net. As shown in Table 8, the experiments begin with the FFB6D baseline and progressively integrate each module.

The introduction of the Pose-Perceptive Convolution (PPC) module yields the most substantial single-component improvement, increasing the ADD-S score by 2.7 points and the VSD score by 9.5 points. This highlights the critical role of PPC in extracting high-quality features for objects with complex geometries. The probabilistic head also provides a significant gain, particularly on the VSD metric with a 7.5 point improvement, which is more sensitive to occlusion and ambiguity. This confirms its effectiveness in enhancing the model’s robustness in cluttered scenes. The SimAM-based Lightweight Fusion Attention (LFA) module offers a stable and effective performance boost on both metrics with negligible computational overhead.

Notably, a strong complementary effect is observed between the modules. For instance, the combination of PPC and the Probabilistic Head results in a substantial 17.5-point gain on VSD, indicating that high-quality geometric features paired with a robust regression mechanism produce a synergistic effect.

Ultimately, the full PPF-Net, which integrates all three components, achieves a total gain of 5.0 points in ADD-S and a remarkable 19.4-point leap in VSD over the FFB6D baseline. This result confirms that the proposed modules are not merely additive but work in concert as an integrated system to build a more accurate and robust pose estimation method.

#### 4.5.2. Analysis of PPC Design

A series of ablation studies are conducted to thoroughly analyze the design of the PPC module and validate its integration strategy. First, the internal components of PPC are evaluated, with results presented in Table 9. Compared to the standard 3 × 3 convolution, the performance improvement from DCN is limited, which suggests that unstructured deformation lacking geometric priors is insufficient for optimally capturing pose-relevant features. PPC-AR, which only adapts the aspect ratio, excels at improving overall geometric alignment accuracy (ADD-S +2.1), while PPC-SD, which only adapts the sampling density, is more effective at enhancing robustness to visible surface discrepancies (VSD +7.3). Finally, the full PPC-Full model, integrating both components, achieves the best performance across all metrics, demonstrating that the combination of structured anisotropy (AR) and adaptive sampling density (SD) is the source of its powerful pose-perceptive capability.

Second, the hierarchical integration strategy of the PPC module is validated, with results shown in Table 10. The data shows that when replacing a single stage, layer3 yields the most significant improvement, confirming its critical role in capturing the overall object contour. Cumulative performance gains are observed as more stages are replaced, with the best results achieved when all three stages, L2-3-4, are equipped with PPC modules. This result demonstrates the necessity of adaptive geometric extraction at each critical phase of feature learning—from parts to the whole—thus establishing the superiority of the chosen integration strategy.

#### 4.5.3. Probabilistic Head and Uncertainty-Weighted Refinement

Table 11 summarizes the ablation study of probabilistic regression and uncertainty-weighted refinement on the MP6D dataset. Replacing the deterministic regression head (L1/SmoothL1, w/o ICP) with a head trained using Gaussian NLL improves ADD-S, from 89.6 to 91.3 (+1.7) and VSD from 71.8 to 79.9 (+8.1). The gain on VSD is consistent with its sensitivity to occlusion and ambiguity.

Furthermore, the predicted variance serves as a reliable signal for guiding subsequent geometric optimization. While adding a standard ICP on top of the probabilistic head further improves performance (ADD-S 92.1; VSD 81.1), the greatest benefit is achieved when the ICP is modified to use $\sigma^{2}$ inverse-variance weighting (ADD-S 92.7; VSD 82.5). This culminates in a total improvement of +3.1 on ADD-S and +10.7 on VSD relative to the deterministic baseline. This demonstrates that the predicted variance, $\sigma^{2}$, is not merely a byproduct of training but a dependable reliability measure for downstream alignment tasks. It enables the explicit suppression of low-confidence correspondences during the refinement stage, leading to more robust results in terms of both visible surface consistency (VSD) and geometric accuracy (ADD-S).

### 4.6. Visualization

**Figure 7:**
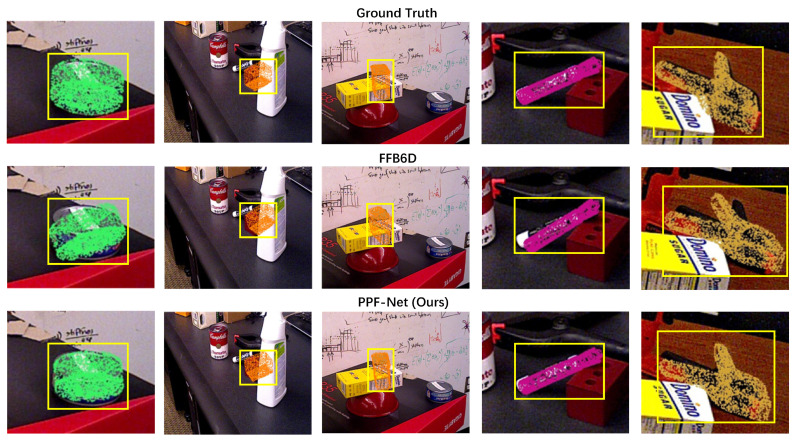
Qualitative comparison of pose estimation results on the YCB-Video dataset.

**Figure 8:**
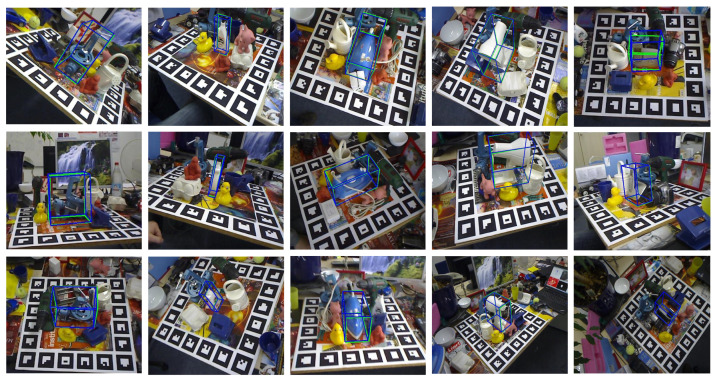
Qualitative results of the proposed PPF-Net on the LINEMOD dataset.

Figure 7 and Figure 8 present the qualitative evaluation results on the YCB-Video and LINEMOD datasets, respectively, to visually validate the effectiveness of PPF-Net. In Figure 7, the prediction results of PPF-Net are compared against the FFB6D baseline and the ground truth, the poses are visualized by transforming the object’s point cloud according to the respective poses and projecting it back onto the image. It can be observed that while FFB6D’s predictions show noticeable deviations when handling symmetric objects like the bowl or objects with challenging shapes like the power drill, PPF-Net consistently maintains a high degree of alignment with the ground truth. This visually demonstrates the superiority of our proposed modules in handling geometric complexity and pose ambiguity.

In Figure 8, the performance of PPF-Net on the classic LINEMOD dataset is further illustrated. Unlike the previous figure, this visualization employs 3D bounding boxes to more clearly illustrate the estimated rotation and translation. The blue bounding boxes represent the pose estimations from PPF-Net, while the green ones correspond to the ground truth annotations. The results show that even for textureless or sparsely textured objects and under various challenging viewpoints, the predicted boxes from PPF-Net maintain a very high consistency with the ground truth boxes, reaffirming its high-precision pose estimation capabilities.

### 4.7. Limitations of the Method

Despite the competitive performance achieved by PPF-Net, the current study has several limitations that provide directions for future work. First, the proposed method, in line with the FFB6D baseline, relies on a predefined set of object keypoints selected offline. Consequently, its performance is sensitive to the quality and selection strategy of these keypoints. Second, while the PPC module effectively handles variations in aspect ratio and scale, it remains a fundamentally local operator. For objects with highly complex topologies or disconnected parts, its ability to model long-range global dependencies might be less effective than methods based on global attention mechanisms like the Transformer. Finally, the current work focuses on instance-level pose estimation, where the object’s CAD model is known during both training and testing. Extending the principles of pose-perceptive feature extraction to these more challenging generalization scenarios presents a promising direction for future research.

## 5. Conclusions

This paper addresses a fundamental issue in 6D object pose estimation: the geometric mismatch between the fixed receptive fields of standard convolutions and the varied projected morphologies of objects. Rather than relying on complex backend fusion modules, we propose a frontend-focused solution centered on the Pose-Perceptive Convolution (PPC). By integrating PPC into the FFB6D baseline, along with a lightweight attention module and a probabilistic regression head, the proposed PPF-Net adaptively aligns feature extraction with object geometry.

Experimental results on multiple benchmarks demonstrate that PPF-Net improves over the baseline and achieves accuracy competitive with recent methods. Although it does not outperform heavy-backend approaches in all metrics, the analysis indicates that it operates with lower computational cost and reduced latency compared to render-and-compare methods. These findings suggest that enhancing frontend geometric perception is a viable strategy for improving robustness, particularly for applications where computational resources are a constraint.

## Figures and Tables

**Table 1:** Conceptual and mechanistic comparison between DCN and PPC.

Aspect	DCN	PPC (Ours)
**Objective**	Feature Sourcing	Geometric Alignment
**Deformation**	Unstructured	Structured
**Adaptivity**	Position	Shape & Scale
**Geometric Prior**	Weak	Strong
**Complexity Analysis (per location for a k×k kernel)**		
**Predicted DoF**	$2k^{2}$ (offsets)	**2** (scales)
**Derived From**	$\Delta p_{n}=\mathrm{Conv}\left(F\right)$	s=SPN(F)

**Table 2:** Quantitative comparison on the MP6D dataset. We report the AUC scores for the ADD-S and VSD metrics without iterative refinement. The best and second-best performances for each object are highlighted in **bold** and underlined, respectively. Results marked with ^†^ are reproduced by us using the official evaluation protocol, while the others are quoted from the corresponding papers. *NR* indicates that the result is not reported under the same setting.

Object	FFB6D ^†^		RCVPose ^†^		GDRNPP ^†^		RDPN6D [30]		CMAGCA [31]		MegaPose ^†^		DFTr [14]		PPF-Net (Ours)	
	**ADD-S**	**VSD**	**ADD-S**	**VSD**	**ADD-S**	**VSD**	**ADD-S**	**VSD**	**ADD-S**	**VSD**	**ADD-S**	**VSD**	**ADD-S**	**VSD**	**ADD-S**	**VSD**
Obj_01	93.3	80.4	94.8	82.3	87.3	74.4	*NR*	*NR*	95.0	*NR*	93.6	**95.7**	**95.4**	94.9	94.2	93.2
Obj_02	92.8	81.5	94.3	83.5	86.8	75.5	*NR*	*NR*	96.1	*NR*	95.5	**94.9**	**96.5**	94.1	93.9	91.5
Obj_03	79.5	43.5	82.5	45.5	73.5	37.5	*NR*	*NR*	**86.1**	*NR*	83.9	**70.4**	84.9	57.4	84.4	58.5
Obj_04	85.0	64.9	88.0	66.9	79.0	58.9	*NR*	*NR*	87.4	*NR*	89.5	**84.2**	**92.0**	80.0	88.6	79.9
Obj_05	76.3	60.2	79.3	62.2	70.3	54.2	*NR*	*NR*	84.3	*NR*	83.2	**75.4**	**86.2**	71.2	84.5	71.3
Obj_06	84.0	63.7	87.0	65.7	78.0	57.7	*NR*	*NR*	95.8	*NR*	95.1	**91.2**	**96.1**	89.0	94.5	87.9
Obj_07	94.9	90.3	96.4	92.3	88.9	84.3	*NR*	*NR*	**97.9**	*NR*	96.5	**98.2**	97.5	97.9	96.9	96.1
Obj_08	89.8	74.7	91.3	76.7	83.8	68.7	*NR*	*NR*	**97.1**	*NR*	95.8	**89.3**	96.8	87.1	96.1	86.2
Obj_09	81.3	46.6	84.3	48.6	75.3	40.6	*NR*	*NR*	**93.0**	*NR*	88.7	**74.4**	91.2	70.2	87.9	70.1
Obj_10	88.9	71.1	90.4	73.1	82.9	65.1	*NR*	*NR*	94.6	*NR*	93.2	**91.7**	**95.0**	90.9	93.9	89.3
Obj_11	84.9	47.1	87.9	49.1	78.9	41.1	*NR*	*NR*	**93.5**	*NR*	89.9	**82.7**	92.4	78.5	90.8	78.7
Obj_12	84.8	54.9	87.8	56.9	78.8	48.9	*NR*	*NR*	**90.1**	*NR*	87.0	**74.2**	90.0	66.2	87.3	68.5
Obj_13	85.4	52.6	88.4	54.6	79.4	46.6	*NR*	*NR*	**96.3**	*NR*	93.2	**87.5**	95.0	85.3	93.0	84.2
Obj_14	88.0	56.2	89.5	58.2	82.0	50.2	*NR*	*NR*	**95.3**	*NR*	92.3	**73.1**	94.1	65.1	93.1	66.9
Obj_15	75.0	13.1	78.0	20.0	69.0	12.0	*NR*	*NR*	83.3	*NR*	84.0	**53.3**	**87.0**	40.3	86.0	45.6
Obj_16	84.0	41.0	87.0	43.0	78.0	35.0	*NR*	*NR*	91.4	*NR*	89.6	**75.3**	**92.1**	71.1	90.6	71.2
Obj_17	93.2	71.2	94.7	73.2	87.2	65.2	*NR*	*NR*	**96.1**	*NR*	92.5	**95.4**	94.3	94.6	93.0	92.9
Obj_18	91.7	81.4	93.2	83.4	85.7	75.4	*NR*	*NR*	**95.9**	*NR*	92.9	**93.3**	94.7	92.5	92.6	90.8
Obj_19	87.3	58.0	90.3	60.0	81.3	52.0	*NR*	*NR*	94.0	*NR*	93.2	**89.4**	**95.0**	87.2	92.9	86.2
Obj_20	85.8	58.5	88.8	60.5	79.8	52.0	*NR*	*NR*	**94.5**	*NR*	91.4	**92.9**	93.9	92.1	92.4	89.0
**ALL (mean)**	86.3	60.5	88.7	62.8	80.0	54.8	**95.9**	*NR*	92.9	*NR*	91.1	**84.1**	93.0	80.3	91.3	79.9

**Table 3:** Quantitative comparison on the YCB-Video Dataset without iterative refinement. All methods are evaluated under the same protocol. The best and second-best performances for each object are highlighted in **bold** and underlined, respectively. Results marked with ^†^ are reproduced by us using the official evaluation protocol, while the others are quoted from the corresponding papers. *NR* indicates that the result is not reported under the same setting.

Method	ADD-S	ADD(-S)
FFB6D [10]	96.6	92.7
DFTr [14]	96.7	94.4
RCVPose ^†^	96.6	95.2
GDRNPP ^†^	96.1	93.6
MegaPose ^†^	97.8	**96.2**
CMAGCA [31]	96.9	94.3
RDPN6D [30]	**98.4**	94.6
PPF-Net (Ours)	96.7	95.4

**Table 4:** Quantitative comparison on the YCB-Video Dataset with iterative refinement (ICP). The best and second-best performances for each object are highlighted in **bold** and underlined, respectively. Methods that do not report ICP-based refinement are marked as NR. Results marked with ^†^ are reproduced by us using the official evaluation protocol, while the others are quoted from the corresponding papers. *NR* indicates that the result is not reported under the same setting.

Method	ADD-S	ADD(-S)
FFB6D + ICP [10]	97.0	93.1
DFTr + ICP [14]	97.3	94.8
RCVPose + ICP ^†^	97.2	95.8
GDRNPP + ICP ^†^	96.6	94.3
MegaPose + ICP ^†^	**98.1**	**96.6**
CMAGCA + ICP	*NR*	*NR*
RDPN6D + ICP	*NR*	*NR*
PPF-Net + ICP (Ours)	97.8	96.0

**Table 5:** Results on the LINEMOD dataset using ADD(-S) @ 0.1d accuracy (%). Symmetric objects (eggbox, glue) are evaluated with ADD-S. All methods are single-view, and results are reported without iterative refinement. Results marked with ^†^ are reproduced by us using the official evaluation protocol, while the others are quoted from the corresponding papers.

LINEMOD	GDRNPP [32]	DFTr [14]	FFB6D [10]	RCVPose [11]	CMAGCA [31]	RDPN6D [30]	MegaPose ^†^	Ours (PPF)
ape	98.0	98.6	98.4	98.5	98.9	99.7	98.6	98.6
can	99.6	100.0	99.8	99.8	100.0	100.0	99.8	99.8
cat	99.0	100.0	99.9	99.9	99.9	100.0	99.9	99.9
driller	99.8	100.0	100.0	100.0	100.0	100.0	100.0	100.0
duck	98.5	99.1	98.4	98.6	99.9	100.0	98.7	98.6
eggbox (S)	100.0	100.0	100.0	100.0	100.0	100.0	100.0	100.0
glue (S)	100.0	100.0	100.0	100.0	100.0	100.0	100.0	100.0
holepuncher	98.9	100.0	99.8	99.8	100.0	100.0	99.9	99.9
**MEAN**	99.2	99.7	99.5	99.6	99.8	99.96	99.6	99.6

**Table 6:** Results on the Occlusion-LINEMOD dataset using ADD(-S) @ 0.1d accuracy (%). The best and second-best results are highlighted in **bold** and underline, respectively. Results marked with ^†^ are reproduced by us using the official evaluation protocol, while the others are quoted from the corresponding papers.

O-LINEMOD	GDRNPP [32]	DFTr [14]	FFB6D [10]	RCVPose [11]	CMAGCA [31]	RDPN6D [30]	MegaPose ^†^	Ours (PPF)
ape	75.5	64.1	47.2	70.1	58.7	64.6	**80.3**	78.6
can	91.3	96.1	85.2	89.5	87.9	**97.0**	92.1	91.8
cat	78.5	52.2	45.7	72.4	57.0	54.8	**81.2**	80.5
driller	86.7	**95.8**	81.4	85.0	88.1	93.1	88.5	87.9
duck	76.2	72.3	53.9	71.8	62.3	68.8	**80.8**	79.2
eggbox (S)	84.5	75.3	70.2	81.3	70.2	78.1	85.6	**86.1**
glue (S)	79.1	73.9	60.1	75.5	78.6	**83.5**	81.0	82.3
holepuncher	89.2	86.8	85.9	88.1	86.1	**96.1**	90.4	90.1
**MEAN**	82.6	77.7	66.2	79.2	73.6	79.5	**85.0**	84.6

**Table 7:** Computational cost comparison profiled on a single NVIDIA A100 GPU (batch = 1). The input resolution is fixed at 480×640 with 12,288 points. FLOPs and Latency represent the end-to-end inference cost for a single object instance. For MegaPose, the metrics account for the official inference pipeline: a coarse network evaluating 50 pose hypotheses followed by a refiner running for 5 iterations, including the rendering overhead.

Method	Params (M)	FLOPs (G)	Peak Mem (GB)	Latency (ms)
FFB6D	33.8	135.2	2.8	28.4
PPF-Net (Ours)	36.5	152.6	3.3	35.7
DFTr	132.4	318.5	5.6	84.2
MegaPose	80.1	1450.5	9.0	165.0

**Table 8:** Component ablation on MP6D. The ADD-S AUC and VSD scores are reported without iterative refinement (ICP). All runs share identical data pre-processing, detector, segmentor, and training schedule with FFB6D. Δ columns show absolute gains over the FFB6D baseline.

Method	PPC	SimAM	Prob. Head	ADD-S AUC ↑	VSD ↑	ΔADD-S	ΔVSD
FFB6D (baseline)				86.3	60.5	–	–
	✓			89.0	70.0	+2.7	+9.5
		✓		86.8	62.0	+0.5	+1.5
			✓	87.2	68.0	+0.9	+7.5
	✓	✓		89.6	71.8	+3.3	+11.3
	✓		✓	90.4	78.0	+4.1	+17.5
		✓	✓	87.8	69.8	+1.5	+9.3
PPF-Net (All)	✓	✓	✓	**91.3**	**79.9**	**+5.0**	**+19.4**

**Table 9:** PPC design ablation on MP6D (w/o ICP). To isolate the convolution-type effect, only 3 × 3 convs in backbone L3–L4 are replaced. PPC-Full outperforms DCN variants, validating that both pose-perceptive anisotropy (AR) and adaptive sampling density (SD) are necessary.

Variant	Description	ADD-S AUC ↑	VSD ↑	ΔADD-S	ΔVSD
3 × 3 Conv	FFB6D standard conv	86.3	60.5	–	–
DCN	Deformable conv	86.8	61.4	+0.5	+0.9
PPC-AR	AR only (fixed density)	88.4	66.4	+2.1	+5.9
PPC-SD	SD only (fixed ratio)	87.2	67.8	+0.9	+7.3
PPC-Full	AR + SD enabled	89.0	70.0	+2.7	+9.5

**Table 10:** Ablation on the integration strategy of PPC modules on the MP6D Dataset. “L2-3-4” denotes replacing the 3 × 3 convolutions in stages layer2, layer3, and layer4 of the ResNet backbone.

Replaced Layers	ADD-S AUC ↑	VSD ↑	ΔADD-S	ΔVSD
None (Baseline)	86.3	60.5	–	–
Only L2	87.1	63.8	+0.8	+3.3
Only L3	87.8	66.5	+1.5	+6.0
Only L4	87.3	64.9	+1.0	+4.4
L2-3	88.4	68.2	+2.1	+7.7
L3-4	88.6	69.1	+2.3	+8.6
L2-3-4 (Full)	**89.0**	**70.0**	**+2.7**	**+9.5**

**Table 11:** Probabilistic regression and uncertainty-weighted refinement on MP6D. We compare deterministic (L1/SmoothL1) vs. probabilistic (Gaussian NLL) heads, and uniform vs. $1/\sigma^{2}$-weighted ICP refinement. The probabilistic head improves both metrics without refinement; $1/\sigma^{2}$-weighted ICP brings further gains, especially on VSD.

Setting	Head/Refinement	ADD-S AUC ↑	VSD ↑	ΔADD-S	ΔVSD
Deterministic	L1/SmoothL1, no ICP	89.6	71.8	–	–
Prob-Head	Gaussian NLL, no ICP	91.3	79.9	+1.7	+8.1
Prob-Head + ICP (uniform)	Prob + ICP (equal weights)	92.1	81.1	+2.5	+9.3
Prob-Head + ICP ($\sigma^{2}$)	Prob + ICP ($1/\sigma^{2}$ weights)	**92.7**	**82.5**	**+3.1**	**+10.7**

## Data Availability

The data presented in this study are available upon request from the corresponding author. All benchmark datasets used in this research can be obtained from their original public sources.
